# SEEDS, simultaneous recordings of high-density EMG and finger joint angles during multiple hand movements

**DOI:** 10.1038/s41597-019-0200-9

**Published:** 2019-09-30

**Authors:** Ana Matran-Fernandez, Itzel Jared Rodríguez Martínez, Riccardo Poli, Christian Cipriani, Luca Citi

**Affiliations:** 1 Brain-Computer Interfaces and Neural Engineering Lab, School of Computer Science and Electronic Engineering, University of Essex, Colchester, UK; 2 The BioRobotics Institute, Scuola Superiore Sant’Anna, Pisa, Italy

**Keywords:** Biomedical engineering, Peripheral nervous system, Electromyography - EMG

## Abstract

We present the SurfacE Electromyographic with hanD kinematicS (SEEDS) database. It contains electromyographic (EMG) signals and hand kinematics recorded from the forearm muscles of 25 non-disabled subjects while performing 13 different movements at normal and slow-paced speeds. EMG signals were recorded with a high-density 126-channel array centered on the extrinsic flexors of the fingers and 8 further electrodes placed on the extrinsic extensor muscles. A data-glove was used to record 18 angles from the joints of the wrist and fingers. The correct synchronisation of the data-glove and the EMG was ascertained and the resulting data were further validated by implementing a simple classification of the movements. These data can be used to test experimental hypotheses regarding EMG and hand kinematics. Our database allows for the extraction of the neural drive as well as performing electrode selection from the high-density EMG signals. Moreover, the hand kinematic signals allow the development of proportional methods of control of the hand in addition to the more traditional movement classification approaches.

## Abstract (summary)

**Table:** 

Measurement(s)	muscle electrophysiology trait
Technology Type(s)	electromyography
Factor Type(s)	age • sex • dominant hand
Sample Characteristic - Organism	Homo sapiens

Machine-accessible metadata file describing the reported data: 10.6084/m9.figshare.9867962

## Background and Summary

For decades, prostheses available to people with hand loss have been either purely cosmetic or allowing a very reduced set of movements, e.g., opening/closing the hand. However, over the last decade, technological advances have allowed the development of small, light-weight prostheses capable of performing a much larger set of movements^1,2^.

While these advanced devices can perform complex movements, generating accurate control signals from biosignals still remains an open issue. The common control strategy consists of using electromyographic (EMG) signals from non-disabled people^3,4^ and amputees^5^ to decode the most useful and/or common types of grasps. Current commercial devices allow rudimentary control of a small number of discrete positions or of a single Degree of Freedom (DoF) using proportional control^6^.

There have been multiple successful attempts at more complex tasks (e.g., direct control of multiple DoF prostheses). However, while these methods report high accuracies in laboratory conditions^7–10^, problems arise when they are exported to daily life circumstances or uncontrolled scenarios, where the error rates often become unacceptable, leading to high abandonment rates or a preference for simpler hook-type hands.

One way to improve the control algorithms and make them less susceptible to scenario changes is to train and test them with larger datasets, increasing the number of subjects and movements, and preferably recording the data under different conditions. However, acquiring reliable datasets is a time-consuming task that limits many studies. This is why some groups are making their datasets available, so other researchers can build/test their work on them.

At the moment, there are three main databases for surface EMG (sEMG) signals recorded from the forearm: Ninapro^11,12^, CapgMyo^13^ and CSL-HDEMG^14^. A comparison of these and our SurfacE EMG ElectroMyoGraphic with hanD kinematicS (SEEDS) dataset^15^ can be found in Table 1. The largest of these is Ninapro, which is in fact an assembly of databases comprising data from non-disabled and amputated volunteers. Most of the databases in Ninapro include kinematics data from the hand. In contrast, CapgMyo and CSL-HDEMG do not include hand kinematics, but sEMG activity is recorded using high-density arrays (HD-sEMG).

**Table 1:** Comparison of currently available datasets and ours.

	Ninapro	CapgMyo	CSL-HDEMG	SEEDS
No. participants	10–40	10–18	5	25
Amputees?	Yes	No	No	No
No. Movements	Up to 53	8, 12, 2	27	13
No. Electrodes	8–16	128 (HD)	192 (HD)	126 (HD) + 8
Sampling rate	100 Hz–2 kHz	1 kHz	2 kHz	2048 Hz
Kinematics?	Data-glove	No	No	Data-glove

For an open dataset, intended to be used for the implementation and testing of different hypotheses and algorithms, we believe that it is important to include the hand kinematics. By including the time series of angles between different joints of the hand, SEEDS allows the development of methods that aim at proportional control of the hand in addition to the more classical approaches based on classification of preset hand grasps. Moreover, the use of HD-sEMG arrays to record muscle activations allows the extraction of the neural drive from the recorded data into motor units^16–19^ and opens up the possibility of exploring muscle synergies and of identifying the optimal locations for electrode placement in amputees.

SEEDS was collected as part of the Dexterous Transradial Osseointegrated Prosthesis with neural control and sensory feedback (DeTOP) project with the aim of helping develop algorithms for proportional control algorithms of robotic hand prostheses. The database contains data acquired from 25 non-disabled participants performing multiple repetitions of 13 different tasks including complex hand movements, common grasps and single finger flexions/extensions. A total of 450 trials are available for each of these movements (=25 participants × 3 sessions/participant × 6 repetitions/movement) in the database. Half of the repetitions were performed at a normal speed and the other half at a slow, controlled pace. We recorded surface EMG by means of a high density array of 126 electrodes placed close to the elbow together with the kinematics of the hand through a data-glove.

We hope that this database will be useful to researchers interested in exploring the relationships between sEMG and hand kinematics in order to create non-invasive, proportionally controlled robotic hand prostheses for people with transradial amputation, which account for the largest population of upper limb amputees^5,20^. Moreover, the placement of the HD-sEMG array allows for the methods developed with this database to be used in both short and long stump scenarios of transradial amputations.

## Methods

### Participants

We collected data from 25 volunteers without any known skeletal and/or neuromuscular disorder (11 males, 14 females; 22 right-handed, 2 left-handed, 1 ambidextrous). Their age was between 21 and 55 years, with mean ± standard deviation = 33.3 ± 9.7 years. Demographic data of each participant are reported in Table 2.

**Table 2:** Demographic data from each participant.

Participant ID	Age	Gender	Dominant Hand
01	30	F	L
02	30	F	R
03	38	M	R
04	29	M	R
05	24	F	R
06	32	M	R
07	34	F	R
08	28	M	R
09	21	M	R
10	55	F	R
11	26	F	R
12	27	F	R
13	29	M	R
14	47	M	R
15	24	F	R
16	46	M	R
17	47	F	R
18	49	F	R
19	29	F	R
20	32	F	L
21	30	F	R
22	43	M	Ambidextrous
23	40	F	R
24	24	M	R
25	18	M	R

Participants were recruited via an email list for the University of Essex with a message that included basic information about the experiment. Upon expressing interest, potential volunteers were sent further details before scheduling a session for the experiment. All candidates agreed to participate and were given a full verbal description of the methods, purpose and protocol for the experiment. They all read and signed an informed consent form before proceeding with the experiment. The experiment was conducted according to the principles expressed in the Declaration of Helsinki and was approved by the Ethics Committee of the University of Essex (United Kingdom) in October 2016.

### Setup for data acquisition

**Fig. 1:**
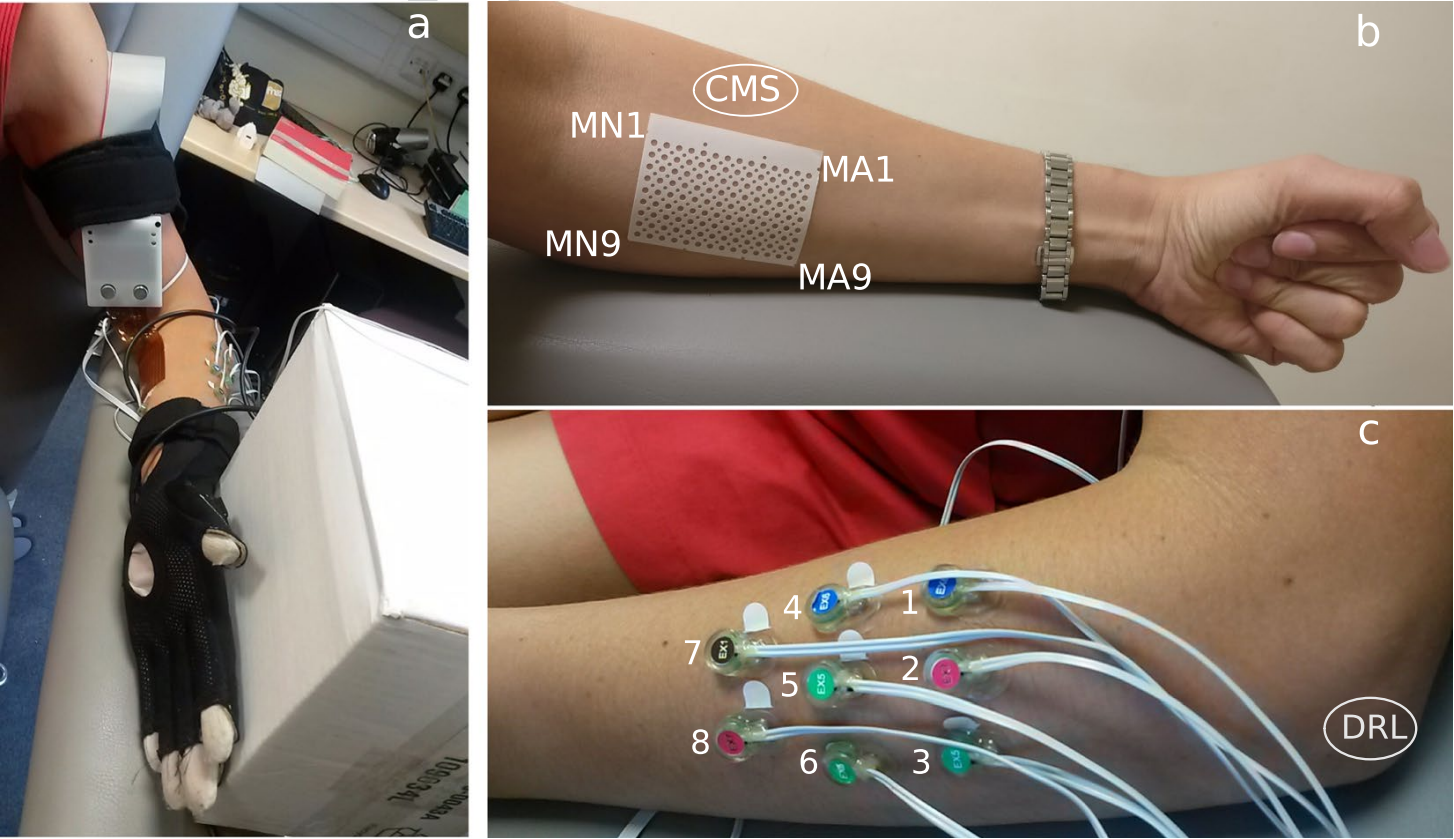
Experimental setup. (**a**) A volunteer wearing the glove, HD-sEMG array, and monopolar EMG electrodes. (**b**) Placement of the HD-sEMG array on the anterior part of the forearm (MA1, MA9, MN1 and MN9 indicate the labels of the electrodes of the corners. The position of the CMS electrode is also labelled in the figure. (**c**) Monopolar electrodes (labelled 1 to 8 in the figure) placed on the extrinsic extensor muscles. The position of the DRL electrode is also labelled in the figure.

The acquisition setup (shown in Fig. 1) was designed to measure hand kinematics simultaneously with muscular activity. In particular, hand kinematics was recorded using an 18-sensor data-glove (CyberGlove III, Cyber Glove Systems LLC, San Jose, CA, www.cyberglovesystems.com) which was connected to an Arduino MEGA ADK. The Arduino was controlled from a laptop through a bespoke GUI (programmed in Visual Studio 2012) which was used to start and stop the streaming of data from the data-glove at the beginning and end of the experiment respectively, and saved the incoming data to a file. The kinematic data recorded with the data-glove corresponded to two bend sensors per finger, four abduction sensors, plus sensors measuring thumb crossover, palm arch, wrist flexion, and wrist abduction that were recorded at a sampling frequency of 60 Hz through the Arduino, which sent a TTL trigger to the EMG amplifier whenever a new sample was acquired for offline synchronisation (further explained on the Data Processing section).

Muscular activity was recorded through a set of 8 monopolar circular EMG electrodes of 4 mm of diameter (BioSemi FLAT Active electrodes, Biosemi, Amsterdam, www.biosemi.com) placed on the posterior part of the forearm of the participant, thus allowing us to measure muscle activations from the extrinsic extensor muscles of the hand and the thumb. These electrodes (shown in Fig. 1(c)) were placed to target the *extensor carpi ulnaris*, *extensor digitorum comunis* and *extensor carpi radialis longus* muscles.

Lastly, a 126-channel HD-sEMG electrode array (circular electrodes of 1 mm of diameter, organised into 9 rows × 14 columns, with 4 mm centre-to-centre distance, covering a surface of 38 × 58 mm^2^ and a registration area = 33 × 53 mm^2^) was used to record the EMG of the anterior part of the forearm, allowing for the measurement of muscle activations from the extrinsic flexors of the hand. The array was positioned to target the belly of the *flexor carpi radialis* muscle.

The placement of the electrodes was performed by asking participants to perform specific movements to isolate the targeted muscles (e.g., for the placement of the HD-sEMG array, volunteers were asked to press against the palm of the experimenter to locate the belly of the *flexor carpi radialis*). Electrodes were positioned where the strongest contraction was found.

All EMG signals were acquired using a BioSemi ActiveTwo EMG amplifier (BioSemi, Amsterdam, www.biosemi.com) through their ActiView software. BioSemi replaces the ground electrodes with two separate electrodes, the Common Mode Sense (CMS) and the Driven Right Leg (DRL). The DRL electrode was placed on the lateral epicondyle of the humerus (Fig. 1(b)). The CMS electrode was placed next to the HD-sEMG array, as shown in Fig. 1(c).

EMG signals were sampled at a rate of 16384 Hz. In order to ensure that the electrodes did not move during the experiment, a latex-free hypo-allergenic elastic band was placed around the forearm of the participant.

Due to equipment restrictions (we only had a left-hand CyberGlove III available), participants wore the HD-sEMG array and the data-glove on their left forearm and hand respectively, regardless of handedness. Participants were comfortably seated on a chair with a straight back and relaxed shoulders in front of a 40-inch LCD monitor on which the instructions and stimuli for the experiment were displayed. Volunteers kept their left arm bent and rested their hand vertically on its ulnar edge on the armrest. Figure 1 shows a diagram of the setup of the sensors on a participant’s arm before placing the elastic band.

### Experimental protocol

Since the data-glove does not record absolute degrees of flexion, but rather an analogue signal that needs to be calibrated so that 0 corresponds to full extension and 1 to full flexion (depending on the joint, this might be up to 90 degrees), the experiment started with a calibration phase in order to obtain the minimum and maximum values to normalize the data from the data-glove. During the calibration phase, participants followed the movements of a hand presented on the screen in front of them.

**Fig. 2:**
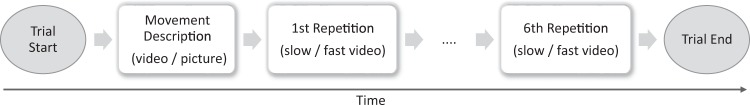
Sequence of a trial. After the description video (in session 1) or image (in subsequent sessions) was shown, participants repeated the movement 6 times at a speed given by the instructions of the screen before moving on to the next movement.

The main part of the experiment consisted of a series of trials, grouped into sessions as described below. The time sequence for a trial is shown in Fig. 2.

A trial started with an explanation of the movement to be performed. In the first session, this consisted of a detailed video which explained the movement and described the correct way of performing it. In subsequent sessions, the explanation was instead in the form of a picture showing the final position of the movement, the required speed (slow or normal) and movement name. In case of doubt, two researchers were in the room with the participant to answer any questions.

After the presentation of this cue, the participant’s task was to repeat the movement six times as indicated by subsequent video stimuli shown on the screen. Each of these repetition videos started with a verbal countdown to give participants time to prepare, followed by the movement being performed either at a slow pace or at a realistic (faster) speed. The order of the speeds was randomly chosen for each participant, session, and movement. The use of the countdown made it easier for the participants to synchronize their movement with the one performed in the video, which they were asked to follow in real time as closely as possible. The glove with markers shown on the videos was intended for a motion capture system used in a pilot version of this experiment. However, due to the long post-processing time required by such system, we decided to use the CyberGlove III instead.

**Fig. 3:**
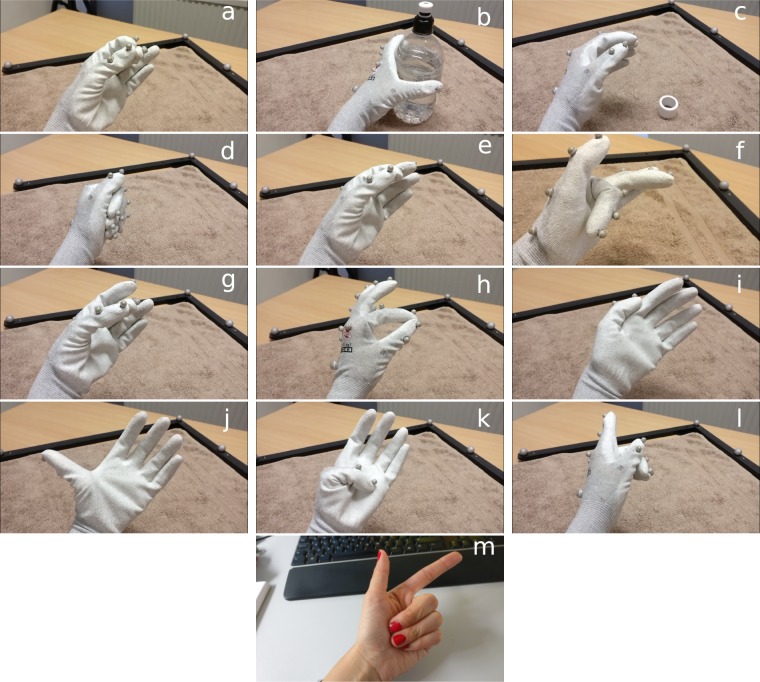
Movements performed. (**a**) three-digit pinch, (**b**) cylinder grasp, (**c**) disc grasp, (**d**) fist, (**e**) index and thumb trumpet test, (**f**) index flexion, (**g**) middle and thumb trumpet test, (**h**) pinch, (**i**) thumb adduction, (**j**) thumb extension, (**k**) thumb flexion, (**l**) point and (**m**) middle, ring and little finger flexion. Note that the difference between the last two is the orientation of the hand and the additional flexion of the thumb in the point movement.

Each session consisted of 13 trials, one for each of the movements shown in Fig. 3, and lasted around 15 minutes (not including breaks). Participants were offered a break between sessions and at the middle of each session (after the first 7 movements), but they were allowed to rest at any other time if they wished to do so.

All participants completed 3 sessions. The order of the movements and the speeds at which they were performed were randomised across volunteers, trials and sessions. Volunteers were asked to always start and finish in the resting position (with the ulnar edge of the hand resting on the armrest and the fingers relaxed).

The movements included pinch and power grasps, flexion and extension of the thumb and index individually, flexion of the middle, ring and little fingers together, and key, disc and cylinder grips (Fig. 3):

- *three-digit pinch*: Bring your index, middle finger, and thumb together as if you were holding a key. Return to the resting position.
- *Cylinder grasp*: Move your hand towards the water bottle, grab and lift it. Then put it back down and return to the resting position.
- *Disc grasp*: Move your hand towards the medical tape roll, rotate your wrist up to lift it (i.e., supination of the forearm), rotate the wrist back down to drop the tape (i.e., pronation of the forearm) and return to the resting position.
- *Fist*: Make a fist with your hand. Return to the resting position.
- *Index and thumb trumpet test*: Bring together the tips of your index and thumb. Return to the resting position.
- *Index flexion*: Bring your index finger towards the palm of your hand. Return to the resting position.
- *Middle and thumb trumpet test*: Bring together the tips of your middle finger and your thumb. Return to the resting position.
- *Pinch*: Bring together the tips of your index and thumb, applying pressure when they touch as if you were holding a piece of paper. Return to the resting position.
- *Thumb adduction*: Press your thumb against the side of your index finger. Return to the resting position.
- *Thumb extension*: Separate your thumb from the rest of your hand. Return to the resting position.
- *Thumb flexion*: Bring your thumb towards the palm of your hand. Return to the resting position.
- *Point*: Pretend to be pointing ahead, by flexing your middle, ring and little fingers together towards the palm of the hand, while extending your index finger and adducting the thumb. Return to the resting position.
- *Middle*, *ring and little finger flexion*: Bring your middle, ring and little fingers together towards the palm of your hand. Return to the resting position.

In the cases where an external object was needed (i.e., in the cylinder and disc grasps, where the participant grabbed and lifted a water bottle and a roll of medical tape, respectively), the object was given to the participant by one of the experimenters at the beginning of the trial, and the participant held it with his/her right arm on the lap.

The calibration and description videos that participants were shown in the first session are available with the data^15^. The slow- and fast-paced videos that volunteers were asked to follow in real time (and which provide the timings for each of the actions described above) are also available with the data.

### Data processing

The kinematic and EMG streams of data were preprocessed individually before synchronising them.

A common average reference was applied to the HD-sEMG signals from the array and to the 8 monopolar signals separately. All EMG recordings were filtered through a zero-phase 3^*rd*^ order band-pass Butterworth filter with cut-off frequencies set at 10 and 500 Hz.

Minimum (*m*) and maximum (*M*) values for each of the signals from the data-glove were extracted from the calibration data for each participant. They were then used to normalise the corresponding kinematic recordings from the experiment (*X*_*orig*_) between 0 and 1, according to the formula:

$${X}_{norm}=({X}_{orig}-m)/(M-m)$$

The minima and maxima for each participant and joint are available with the dataset^15^, so that other researchers can study the global movements of the hand instead of the individual joint movements.

The synchronisation of the two data streams (one from the data-glove and the other from the ActiveTwo) was performed offline. For this, we used the TTL trigger signal from the Arduino ADK that had been recorded by the ActiveTwo system together with the EMG data, and placed each sample at the time point where the trigger input changed value, before upsampling the data from the data-glove to the sampling frequency of the EMG.

To reduce space requirements, as we will explain below, each of the repetitions of each trial was extracted and stored separately for the database.

For the database, the EMG stream was downsampled to 2048 Hz and the data-glove to 256 Hz. The raw streams of data at the original sampling rates are available from the authors upon request.

## Data Records

The data produced during the described experiment and methods are freely accessible and may be downloaded from^15^, which is a general-purpose repository that makes research outputs available in a citable, shareable and discoverable manner. The format and content for the dataset is described below.

For each participant, the database contains a folder with 234 (=13 movements × 3 sessions × 6 repetitions) files in Matlab format (.mat file extension): one per session, movement and repetition. In turn, each of the mat files contains a set of variables, namely:

- subject: subject number;
- session: session number;
- date: date in which the data were collected, in format YYYYMMDD;
- movement: name of the movement performed by the subject;
- fs_emg: sampling frequency of the EMG recordings (2048 Hz);
- fs_glove: sampling frequency of the data-glove signals (256 Hz);
- speed: speed at which the movement was performed (fast or slow);
- emg: 2D array (channels × samples) of sEMG signals. This array contains 134 rows, each one representing a recording site. The first 126 rows belong to the HD-sEMG array, the remaining ones are from the individual sEMG sensors placed on the posterior part of the forearm;
- channels_emg: contains the names of the channels for the EMG signals in the order as they appear on the data matrix (see Fig. 1 (right) for the location of each channel on the participant’s forearm);
- glove: 2D array (channels × samples) of signals from the 18 sensors (one per row) of the data-glove. The signals represent the normalised angle of each joint and have been normalised to be between 0–1;
- channels_glove: contains the names of the channels for the data-glove in the order as they appear on the data matrix.

Each filename has the format *detop_exp01_subjAA_SessB_CC_DD*.*mat*, where *AA* is the subject number (taking values between 1–25), *B* the session (1–3), *CC* the movement (1–13) and *DD* the repetition number (1–6). The prefix detop refers to the fact that this database was collected as part of the DeTOP European project.

An additional .mat file (*subjAA_calibration_values*.*mat*) is provided for each participant specifying the minimum and maximum values used for each of the joints used for normalisation (see Section “Data Processing”). These files contain the following variables:

- calibration_min: 1D array of 18 numbers, each detailing the minimum value for each of the joints of the dataglove, in the same order defined by the channels_glove field of the mat files described above.
- calibration_max: 1D array of 18 numbers, each of them being the maximum value for each of the joints of the dataglove.

We also provide some functions in MatLab and Python to work with the data. These are in a separate folder called *Functions* in^15^. The function resample_glove may be used to upsample the glove matrix to be of the same number of samples as the EMG data provided without losing the synchronisation between the two arrays of data.

An additional function, load_data, together with a short use example, is provided in Python to load data from specific participants, sessions or movements, or a given number of repetitions.

## Technical Validation

The quality of each EMG signal was assessed when placing the electrodes by measuring the voltage offsets from BioSemi’s ActiView. During data acquisition, artefacts in the EMG signals were continually monitored. Behavioural data from the participants, where relevant (e.g., a volunteer not complying with instructions properly), were recorded and can also be found in^15^.

### Synchronisation of signals

**Fig. 4:**
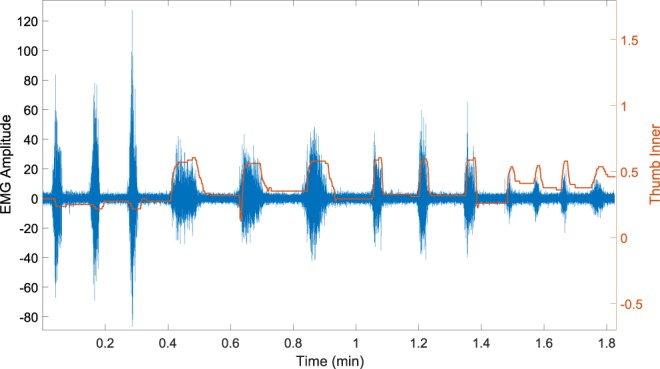
Synchronisation between EMG and data-glove. Example of synchronisation between an EMG channel (blue) and the thumb inner sensor of the glove (orange). The movements correspond to the last three repetitions of middle finger flexion (session two, fast), all six of point (session two, slow and fast) and the first four of middle and thumb trumpet (session two, three fast and one slow) of participant 03.

We started the validation of the dataset by ensuring that the synchronisation from the EMG signals and the data-glove were adequate. Figure 4 shows an example of the synchronisation between the data-glove and EMG streams across multiple repetitions of different movements using data from one EMG channel from the HD-sEMG array and the thumb inner sensor from the data-glove.

### Repeatability

**Fig. 5:**
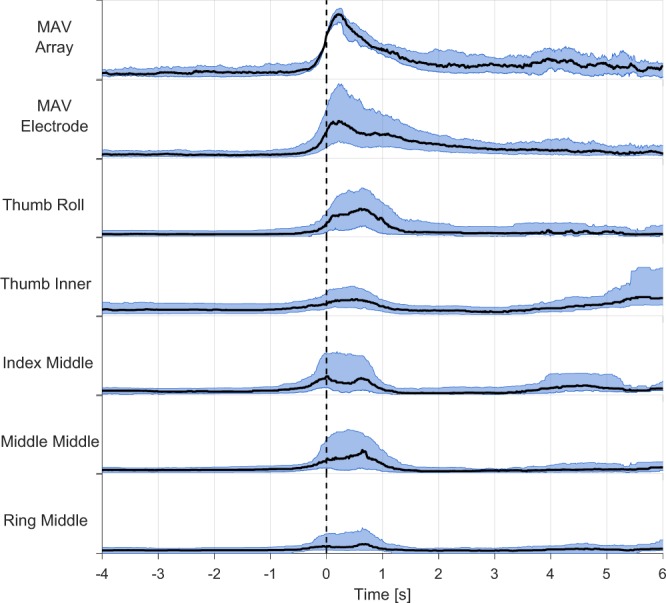
Repeatability of the middle, ring and little finger flexion movement at fast speed. The first row corresponds to the mean of the MAV of the six central channels of the HD-sEMG array, the second row to the MAV of the fifth EMG electrode and the rest of the rows belong to the dataglove sensors. For all rows, the vertical axis is in the range between zero and one, the black line represents the media and the blue ones mark the interquartile range.

To assert the repeatability of the movements and signals and the absence of fatigue effects, the data from all subjects, sessions and repetitions were aligned and plotted separately for each movement. The EMG signals were normalised (between 0 and 1) by the maximum value of the Mean Absolute Value (MAV) feature per subject. This normalization was made individually for the array and each of the electrodes, with its respective maxima. In order to account for delays between the instructions and the movement execution, to synchronise the beginning of the repetition across all users we set a threshold at 0.5 of the MAV of the median of the six central channels of the HD-sEMG array, calculated with sliding windows of 120 ms and steps of 10 ms. This graphical representation was created for each of the 13 movements at each of both speeds and these figures are included on the repository. Figure 5 shows an example of this for the middle, ring and little finger flexion movement.

### Movement classification

To verify that the data allow the recognition of hand movements, we applied a simple K-Nearest Neighbours (KNN) classifier (with *k* = 5) on features extracted from the data and compared its performance with chance accuracy.

We checked the classification performance of a KNN classifier on the 13 movements from our database individually for each participant. For this, we first segmented the data using a sliding window of 200 ms and a 25% overlap^11,21^. This allowed us to check the accuracy of the classifier using trials of different length. Moreover, since the beginning of each repetition started with a countdown, we also considered the impact of cropping different lengths at the beginning of the trial.

To reduce the number of features, we used only half of the electrodes from the HD-sEMG array. For each electrode and window we extracted 5 features:MAV, variance, Root Mean Square (RMS), number of zero crossings and Average Amplitude Change (AAC)^21,22^.

**Fig. 6:**
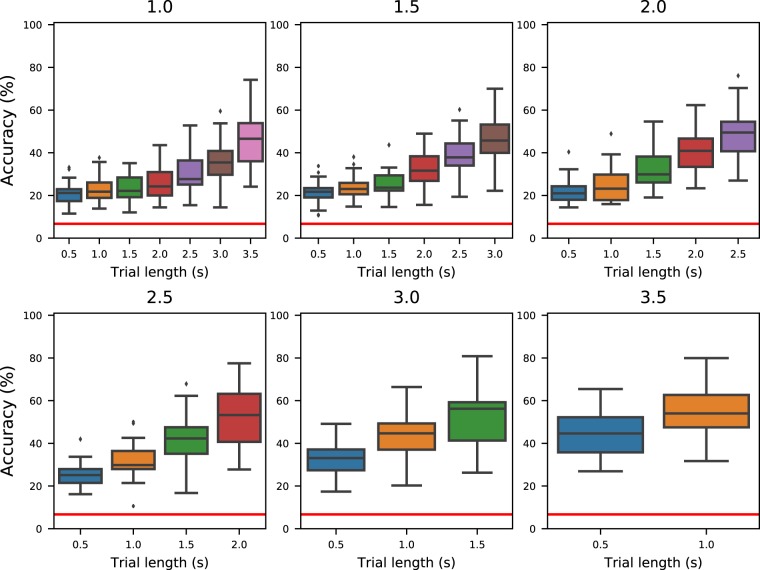
Average accuracies for fast-type trials. Distribution of average accuracies across all participants for the trials of type “fast” as a function of the trial length considered. Each subplot contains the results for a different cropping length at the beginning of the trial.

**Fig. 7:**
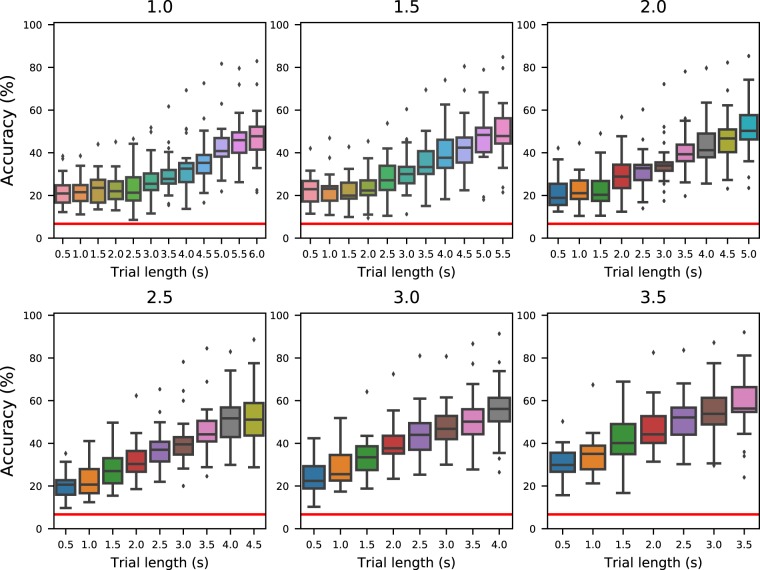
Average accuracies for slow-type trials. Distribution of average accuracies across all participants for the trials of type “slow” as a function of the trial length considered (x-axis) and the length of the cropped period at the beginning of the trial.

For each combination of cropping length (between 1 second and 3.5 seconds, increased in intervals of 500 ms) and trial length (from 500 ms to a maximum of 6 s, depending on the type of trial and the length of the cropped interval, increasing in intervals of 500 ms), we performed 10-fold cross-validation for each participant. The accuracies are reported in Fig. 6 for the trials of type “fast” and Fig. 7 for the trials of type “slow”. The chance accuracy was measured to be 6.7 ± 7.4% (mean ± standard deviation). In all cases, the average accuracy of cross-validation is above this range, marked by a horizontal red line in both figures. We acknowledge that the results are well below the state of the art. This is due to the fact that no efforts were made to optimise/select the input features as well as the classifier, since the main objective was to verify that the presented signals are not just noise but informative data.

## Usage Notes

There are several potential uses for this database. The main one is the application of pattern recognition and machine learning methods to recognize movements from sEMG signals in order to compare different signal features and methods. Since our dataset also contains the kinematics of each digit of the hand, proportional control methods may also be studied using our data.

We encourage any use that can contribute to the creation of naturally controlled robotic hand prostheses for individuals with transradial amputation. Moreover, since the sEMG array was placed very close to the elbow, our dataset also allows for the development of control algorithms for short residuum amputees. Our database can additionally be used to study hand kinematics and dynamics in intact subjects.

In any use involving the data from the CyberGlove III, the users should bear in mind that the calibration phase performed at the beginning of the experiment did not cover all joints. For this reason, the normalised angles stored in the structure occasionally fall out of the range 0–1. However, given that we provide the values used for normalisation, it is easy to reconstruct the original values measured by the CyberGlove III. Moreover, this will also allow for the study of the global movements of the hand, rather than individual joint movements.

## Data Availability

The preprocessing and normalisation of the EMG and kinematics data, as well as the synchronisation between them were performed using custom Python code, which can be obtained from the authors upon request. The Python script used for the classification of movements (see the Technical Validation section) can be found in a folder called functions.
